# Diagnosis of depression based on facial multimodal data

**DOI:** 10.3389/fpsyt.2025.1508772

**Published:** 2025-01-28

**Authors:** Nani Jin, Renjia Ye, Peng Li

**Affiliations:** ^1^ Materdicine Lab, School of Life Sciences, Shanghai University, Shanghai, China; ^2^ Research Department, Third Xiangya Hospital of Central South University, Changsha, China

**Keywords:** depression, multi-modal data, feature fusion, spatial-temporal attention, artificial intelligence

## Abstract

**Introduction:**

Depression is a serious mental health disease. Traditional scale-based depression diagnosis methods often have problems of strong subjectivity and high misdiagnosis rate, so it is particularly important to develop automatic diagnostic tools based on objective indicators.

**Methods:**

This study proposes a deep learning method that fuses multimodal data to automatically diagnose depression using facial video and audio data. We use spatiotemporal attention module to enhance the extraction of visual features and combine the Graph Convolutional Network (GCN) and the Long and Short Term Memory (LSTM) to analyze the audio features. Through the multi-modal feature fusion, the model can effectively capture different feature patterns related to depression.

**Results:**

We conduct extensive experiments on the publicly available clinical dataset, the Extended Distress Analysis Interview Corpus (E-DAIC). The experimental results show that we achieve robust accuracy on the E-DAIC dataset, with a Mean Absolute Error (MAE) of 3.51 in estimating PHQ-8 scores from recorded interviews.

**Discussion:**

Compared with existing methods, our model shows excellent performance in multi-modal information fusion, which is suitable for early evaluation of depression.

## 1 Introduction

Depression, also known as clinical depression or depressive disorder, is a prevalent and serious mental health condition that manifests through persistent low mood, lack of energy, and other symptoms that significantly impact an individual’s thoughts, emotions, behaviors, and overall health (1). According to the World Health Organization, approximately 280 million people worldwide suffer from depression, with 15% of those affected eventually dying by suicide (2). The multifaceted nature of depression, influenced by social, psychological, and biological factors, underscores the necessity for a comprehensive approach to its treatment (3). Long-term stress, genetic predispositions, and adverse social environments are key contributors to the onset of depression, necessitating multifaceted treatment strategies to help patients regain a healthy life.

Traditional methods for diagnosing depression often rely on clinical evaluations by doctors and self-reports from patients. These scale-based methods are fraught with challenges such as high subjectivity, potential misdiagnosis, regional disparities, and a general lack of medical awareness. Moreover, the subtle nature of depressive symptoms means many individuals fail to recognize their condition promptly, leading to delayed treatment and worsening symptoms. Therefore, developing auxiliary diagnostic tools based on objective indicators is crucial for improving early diagnosis and treatment outcomes.

Recent advancements in artificial intelligence (AI) and deep learning have introduced new possibilities for assisting in the diagnosis of depression. These technologies have shown promise in identifying patterns and features indicative of depression through various data modalities (4). However, there are still some limitations in the research aimed at automatic diagnosis of depression. Some studies only consider global features and ignore local features in facial video data, which may lead to insufficient capture of subtle facial changes related to depression. Other studies only consider video data without combining audio information, ignoring the importance of multimodal information. In addition, the design of some models is too complex, which leads to the poor interpretability of the model and the difficulty in understanding its inner mechanism. We address the limitations of previous related work and propose a novel multimodal deep convolutional network, aiming to overcome these problems and provide a more efficient solution for the automatic diagnosis of depression.

In this study, we propose a novel deep learning approach that leverages multimodal data fusion to automatically diagnose depression using facial video and audio data. Our method enhances the extraction of visual features through a spatiotemporal attention module and combines Graph Convolutional Networks (GCN) and Long Short-Term Memory (LSTM) networks to analyze audio features. By integrating these multimodal features, our model effectively captures diverse patterns associated with depression. Our experimental results demonstrate that the proposed method outperforms existing approaches in terms of performance metrics, making it a promising tool for the early evaluation and diagnosis of depression. The main contributions of our study are as follows:

1. We introduce a novel multimodal network architecture that comprehensively integrates video and audio information, significantly enhancing the reliability of depression diagnosis.
2. We design a feature fusion model that effectively combines temporal and spatial features, providing a more comprehensive representation of video data and facilitating a deeper analysis of the patient’s psychological state.
3. We employ a combined GCN and LSTM model to process audio data, constructing a graph structure to analyze Mel-Frequency Cepstral Coefficients (MFCC), thereby improving the interpretability and accuracy of the diagnostic process.

This paper is organized into five sections. The first section presents the research background, discusses the status and challenges of depression identification, and introduces the objectives and significance of the study. The second section reviews recent methods for depression evaluation using both single-modal and multi-modal data. The third section details the proposed method, including the overall network architecture and its components. Section four includes the experimental environment, training process, dataset details, results and discussions. Finally, section five summarizes the contributions of this study, evaluates the advantages and limitations of the proposed method, and outlines directions for future research.

## 2 Related work

Studies have shown that depression state is closely related to patients’ head and face activities (5). Currently, some studies have tried multi-modal fusion of facial video information and other types of data, such as voice features and text information, to improve the accuracy of depression diagnosis. By utilizing multiple sources of information, the emotional state and psychological characteristics of patients can be captured more comprehensively, leading to more accurate assessment.

Al Jazaery and Guo (6) used 3D convolutional neural networks to extract deep spatio-temporal features of closely cropped aligned facial regions and relatively large head regions respectively, and then used recurrent neural networks to continue learning spatio-temporal information for final prediction. It is the first application of 3D convolutional neural networks to depression level analysis and shows great promise. But it focuses more on visual-based non-verbal data and does not take audio into account. Sun Haohao et al. (7) performed face detection, alignment and cropping on video frames in AVEC2013 (8) and AVEC2014 (9) depression databases to obtain the whole face image and the local eye and mouth region. Then, the deep convolutional neural network that fuses the attention mechanism of the channel layer is used to extract the corresponding global features and local features. The multiple visual features learned are more discriminative than the global features alone. But this study does not consider the influence of the audio. Yuchen Pan et al. (10) proposed the Spatio-Temporal Attention Depression Recognition Network (STA-DRN), which mainly uses the spatio-temporal attention (STA) mechanism to generate spatial and temporal attention vectors, so as to capture the global and local spatio-temporal relationships of features. In the STA module, there is also an attention vector fusion strategy that fuses spatial and temporal domain information. This model can capture the dynamic change process of facial expression and enhance the feature correlation in the process of depression recognition. JH Kim et al. (11) introduces the customized VGG-19 (CVGG-19) architecture, which integrates designs from VGG, Inception-v1, ResNet, and Xception to enhance facial emotion recognition (FER). The CVGG-19 significantly improves performance by 59.29% and reduces computational cost by 89.5% compared to the original VGG-19. Additionally, CVGG-19 achieves an average F1-score that is 3.86% higher than Inception-V1, ResNet50, and Xception architectures. Constantino Álvarez Casado et al. (12) extracted remote photoplethysmography (rPPG) signals directly from facial videos and computed a variety of statistical, geometric and physiological features including heart rate. These features were fed into machine learning regression models to identify different levels of depression. The results of this approach are comparable to other audiovisual models based on voice or facial expression.

Some studies only focus on audio information for depression recognition. Momoko Ishimaru et al. (13) input the feature vector converted from audio data into graph convolution layer and dense layer in turn, and finally obtain the prediction score. This new regression model uses the generated graph-structured data to express correlations between audio features, which can be exploited to assess the severity of depression. Li et al. (14) built speech signals into speech graphs based on feature similarity to input Graph-LSTM neural network for classification. The network is a new graph neural network structure combining LSTM aggregator and weighted pool, which enhances the interpretability of the model and can effectively identify speech emotional features. However, the model also has the shortcomings of redundant speech graph features and lack of visual features.

Some advancements in bimodal speech emotion recognition (SER) using both acoustic and text data, focusing on the significance of attention mechanisms and fusion strategies in combination with traditional deep learning techniques. Also there are some challenges such as limited datasets and difficulties in data acquisition (15).

Uddin et al. (16) input the preprocessed audio clips and video clips into the spatio-temporal network based on audio and video. The dynamic feature descriptor Volume Local Directional Structural Pattern is introduced to encode the structure, so as to extract the dynamic facial features. Then, Temporal Attentive Pooling is used to summarize the segmentation features, and Multi-modal factorized bilinear pooling is used to fuse the multimodal features. Finally, the corresponding BDI-II scores were obtained by regression to estimate the severity of depression. This method has strong feature extraction ability and considers multi-modal data but ignores the association between high-level semantic features and channels. Ming Fang et al. (17) comprehensively considered video, audio and text information, and designed a multi-modal fusion model with multi-level attention mechanism (MFM-Att) for depression detection. The model uses two LSTMs to learn video and audio features, and a Bi-LSTM with attention mechanism to learn text features, and then inputs these three outputs into the MFM-Att for feature fusion. This design can make information complementary between different modalities. However, the complexity of the model needs to be improved.

Improving the interpretability of diagnostic models for depression is crucial for clinical practice. David Gimeno-Gomez et al. (18) present a simple and flexible multimodal temporal model capable of recognizing nonverbal cues to depression from noisy real-world videos. They visualize the level to which these features contribute to the results through integrated gradients (19) based on audio-speech embeddings, facial emotion embeddings, facial, body and hand signatures, as well as gaze and blink information.

## 3 Methods

**Figure 1:**
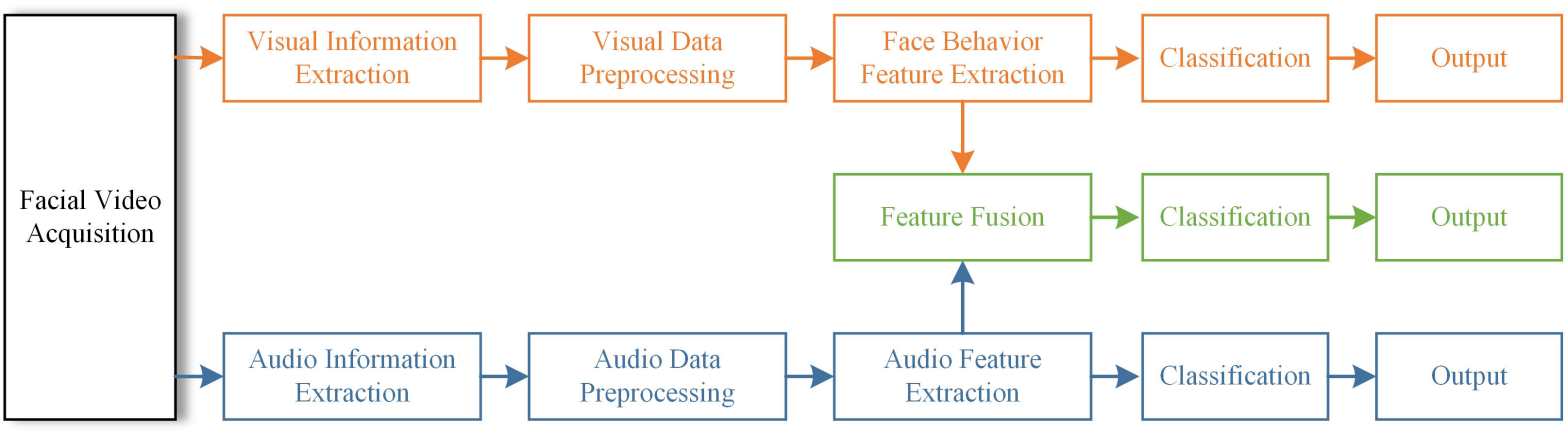
Framework for depression diagnosis.

**Figure 1** shows the framework of the proposed method for diagnosing depression based on multimodal data. Firstly, visual information and audio information are extracted from the recorded videos of the participants, and the two kinds of information are pretreated separately. Then, the feature extraction is performed on the preprocessed data and the multimodal feature set is obtained by feature fusion. After that, the processed features are classified and the respective classification results are output. This framework allows the model to synthesize visual and audio information, which helps to deeply mine the hidden information in the data. In the process of facial behavior feature extraction, we use the spatio-temporal attention module to strengthen the correlation between features and video frames. For audio features, GCN and LSTM are mainly used.

### 3.1 Visual feature extraction

In order to effectively extract information from the facial behavior features, we propose Temporal-Spatial Network for Depression Diagnosis (TSNet-DD). The proposed model incorporates a temporal attention module and a spatial attention module to capture global and local features at the temporal and spatial levels from video frames. The core of TSNet-DD is that it can use the Temporal-Spatial Attention Module (TSAM) to enhance the correlation between pixels and frames.

**Figure 2:**
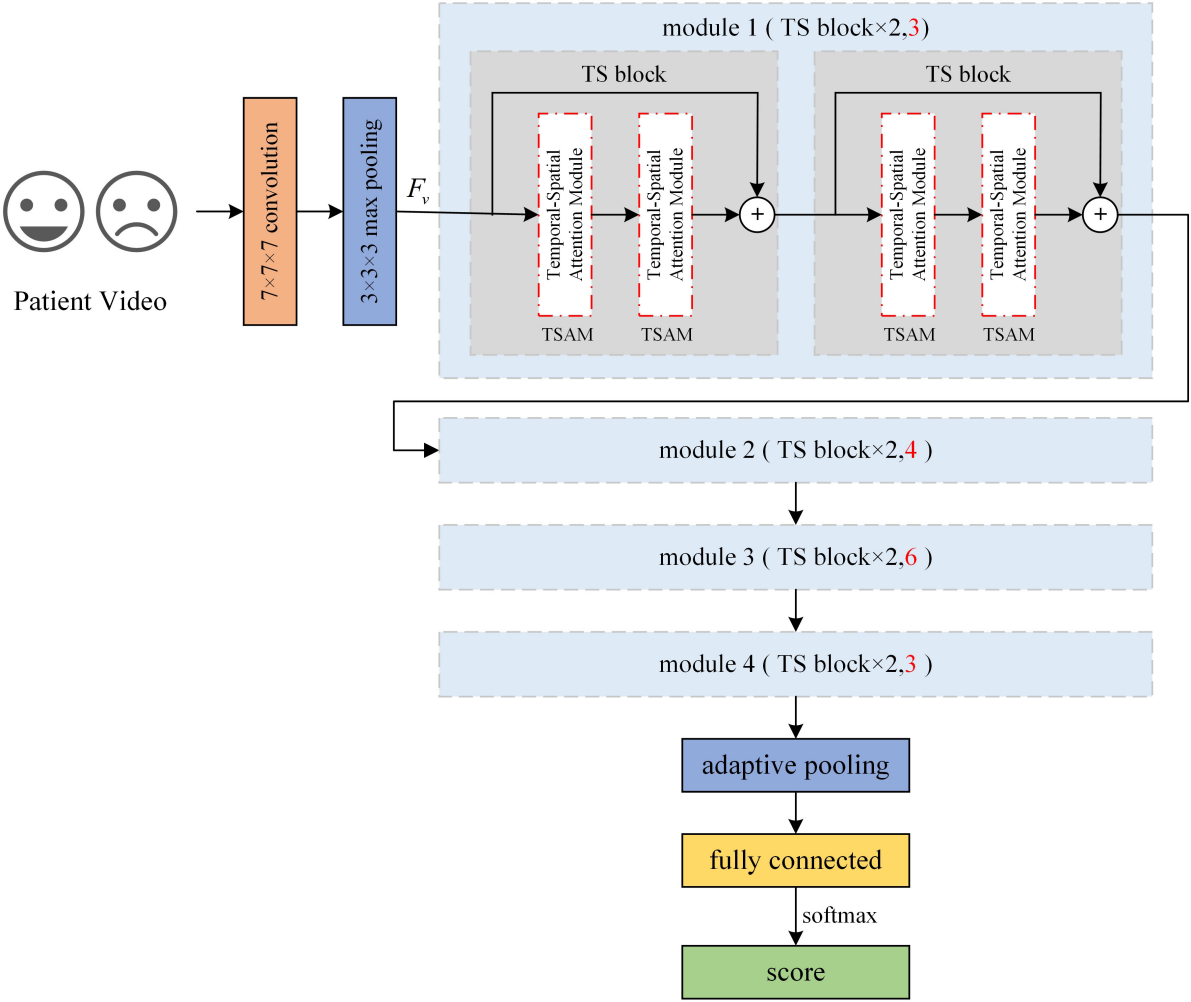
Architecture of TSNet-DD.

The overall architecture of TSNet-DD network is shown in **Figure 2**. The initial layer of the network uses a 7×7×7 convolution kernel to perform downsampling with a step size of 1×2×2 to extract low-level features of the input image. Next, a 3×3×3 pooling operation with a step size of 1×2×2 is performed in the second layer, and the resulting features are denoted as $F_{v}$ . The subsequent module1, module2, module3 and module4 correspond to different convolutional layers in the ResNet, and each module consists of a different number of residual blocks. TSNet32-DD corresponds to the ResNet18, that is, each module contains two residual blocks, and two sub-modules are also contained within each residual block. In ResNet18, these submodules are 3×3 convolutional layers, while in our network, we introduce TSAM. Therefore, a total of 32 TSAMs are used in the ResNet18-based network, and we refer to this network as TSNet32-DD.

Similarly, in the ResNet34-based network, there are 3,4,6 and 3 residual blocks in each module (sections marked red in **Figure 2**). Each residual block still contains two TSAMs, resulting in a total of 64 TSAMs in the final network, thus this network is called TSnet64-DD. After all residual modules, a pooling operation is performed on the feature map to resample the features into fixed shapes, and finally a fully connected layer is used to classify the subjects.

**Figure 3:**
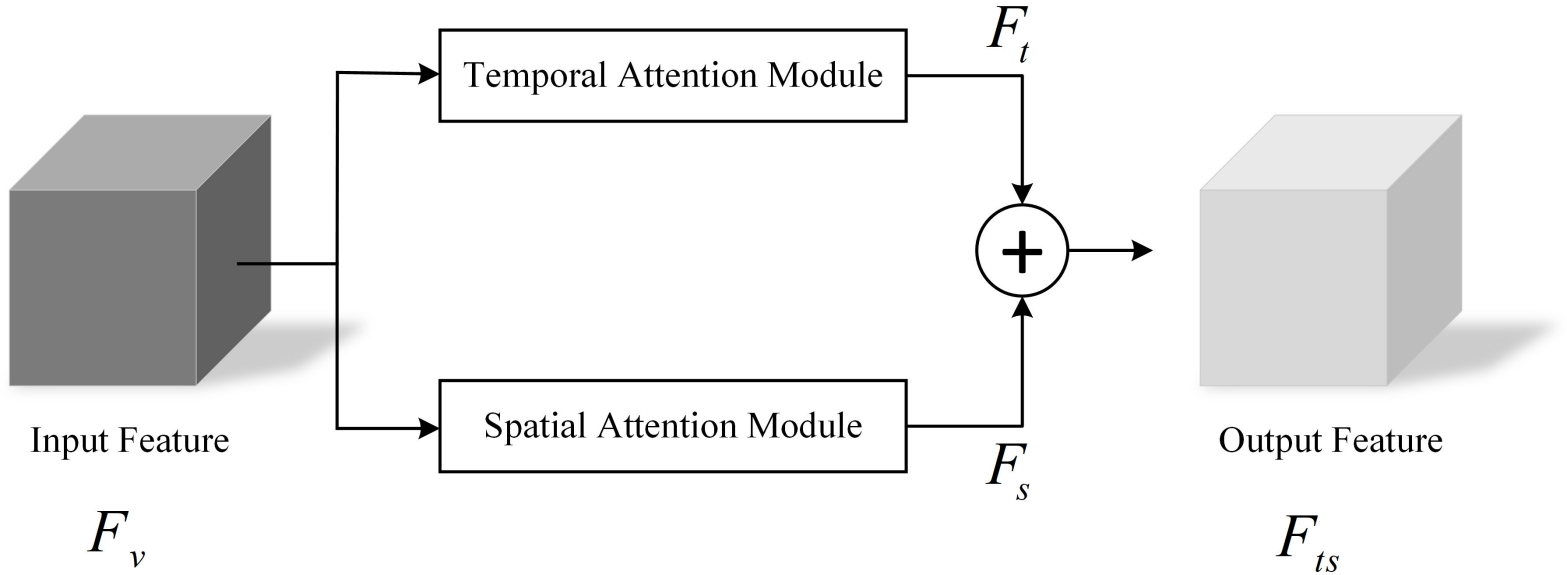
Temporal-spatial attention mechanism.

The feature extraction module TSAM in TSNet-DD contains a temporal attention module and a spatial attention module. These two modules are used to generate the temporal attention weight vector $W_{t}$ and spatial attention weight vector $W_{s}$ of the input $F_{v}$ , respectively, so as to obtain the corresponding temporal feature $F_{t}$ and spatial feature $F_{s}$ . Then, these two kinds of features are fused to capture the intrinsic relationship between spatial-temporal features, assigning adaptive weights to the features with spatio-temporal information. The structure of TSAM is shown in **Figure 3**.

The fusion process of temporal attention module and spatial attention module could be expressed by the following formula:


(1) $$F_{ts}=F_{t}+F_{s}$$

#### 3.1.1 Temporal attention module

**Figure 4:**
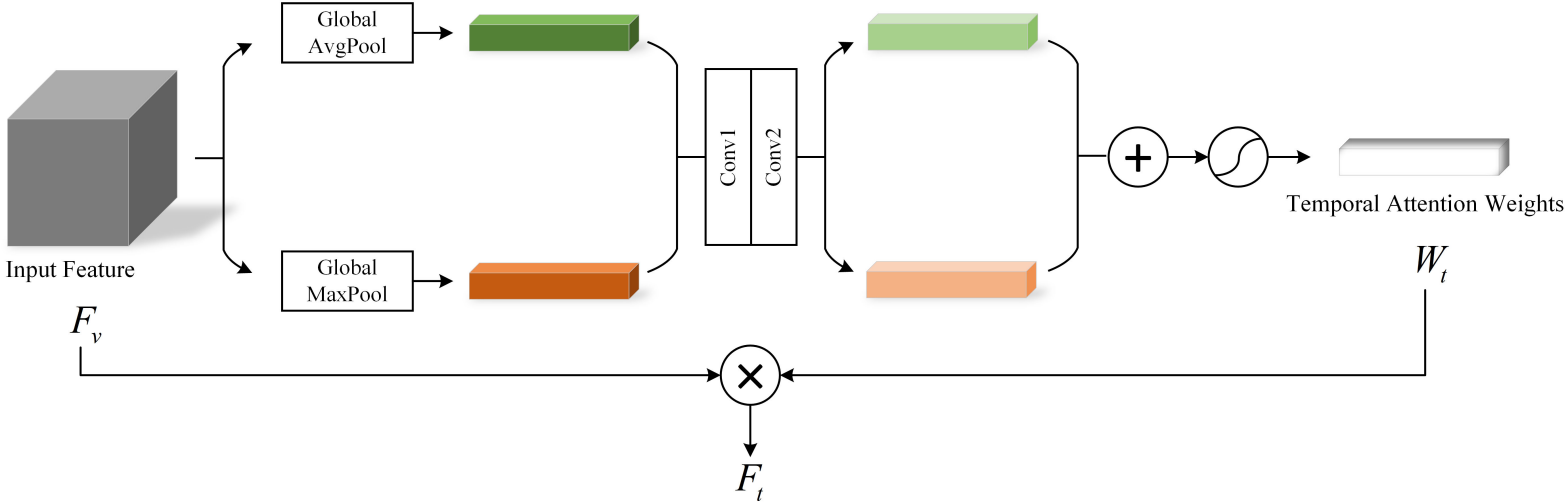
Temporal attention module.

For video data of patients with depression, intra-frame temporal changes are crucial for depression recognition. Such temporal changes can be short term or long term dynamics spanning several seconds. Although short-term features could capture dynamic information between several frames, their ability to extract long-term dynamic features is limited. To address this problem, we introduce a temporal Attention module (TAM) for enhancing temporal information. The specific structure of this module is shown in **Figure 4**.

In the TSnet-DD model, after the second layer of 3×3×3 pooling operation, the feature $F_{v}$ is obtained, and its size is assumed to be H×W×C. The feature $F_{v}$ is sent to the TAM. Firstly, the global average pooling and max pooling operations are performed respectively to obtain two 1×1×C channel descriptions. Subsequently, these two descriptions are fed into a two-layer neural network with shared weights for processing. Then the resulting two features are added and the weight coefficient $W_{t}$ is obtained through the sigmoid activation function. Finally, $W_{t}$ is multiplied with the original input feature $F_{v}$ to obtain the new scaled feature $F_{t}$ . The process of TAM can be summarized as follows:


(2) $$W_{t}=\sigma \left(Conv\left(AvgPool\left(F_{v}\right)\right)+Conv\left(MaxPool\left(F_{v}\right)\right)\right)$$

(3) $$F_{t}=F_{v}*W_{t}$$

To further visualize the architecture and data transfer process of TAM, we show its pseudo-code in **Algorithm 1**.

Algorithm 1 **Pseudocode of temporal attention module.**

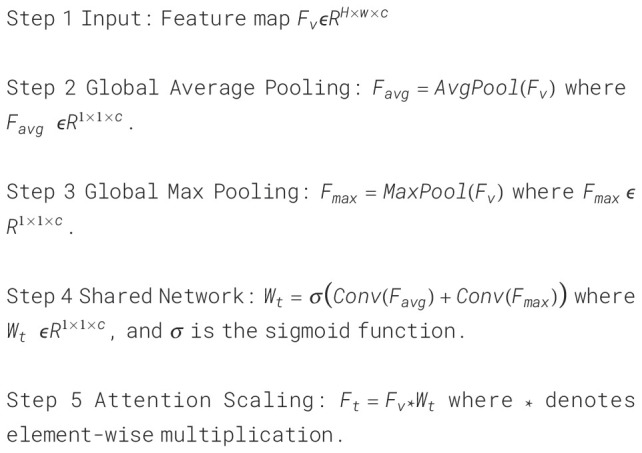

#### 3.1.2 Spatial attention module

**Figure 5:**
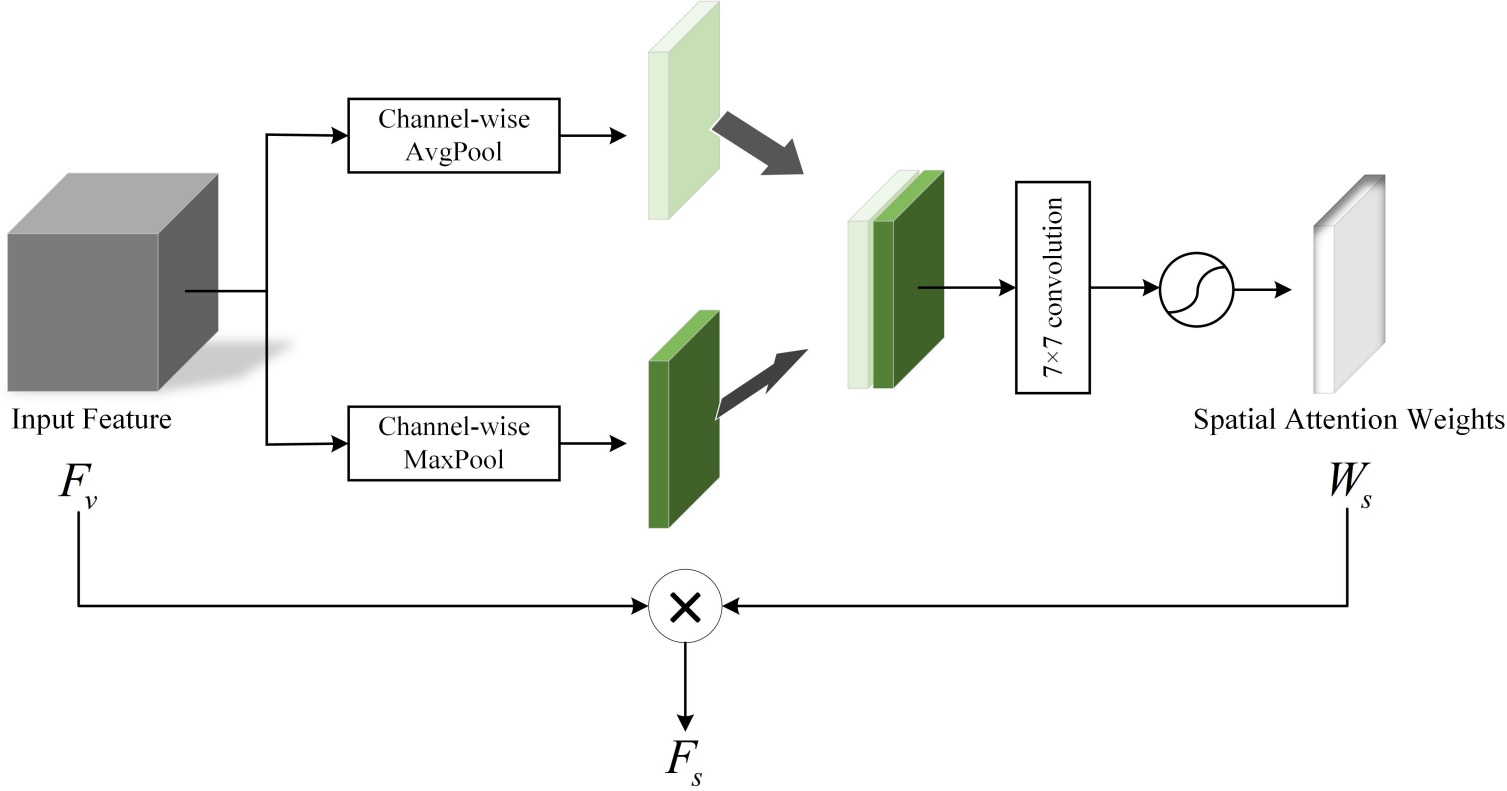
Spatial Attention Module.

In our collection of videos about people with depression, some useful features usually appear in a sequence of consecutive video frames. Therefore, whether features can identify spatial order information is crucial in depression diagnosis. In addition, different locations of the face have their own unique features, and there are subtle relationships between these location features that cannot be captured by our naked eyes (20). With this in mind, we employ a Spatial Attention Module (SAM) to generate spatial vectors to capture the spatial information. The structure of SAM is shown in **Figure 5**.

In SAM, the input feature $F_{v}$ can determine where the features are meaningful. Firstly, the average pooling and maximum pooling of the channel dimension are performed on $F_{v}$ respectively to obtain two channel descriptions of size H×W×1, then the two descriptions are concatenated together in the channel dimension. Next, through a 7×7 convolutional layer and the activation function sigmoid, the weight coefficient $W_{s}$ are obtained. Finally, the $W_{s}$ is multiplied with the input feature $F_{v}$ to obtain the final spatial attention vector $F_{s}$ . This process can be expressed as follows:


(4) $$W_{s}=\sigma \left(Conv\left(\left[AvgPool\left(F_{v}\right),MaxPool\left(F_{v}\right)\right]\right)\right)$$

(5) $$F_{s}=F_{v}*W_{s}$$

The flow of the Spatial Attention Module is shown in **Algorithm 2**.

Algorithm 2 **Pseudocode of spatial attention module.**

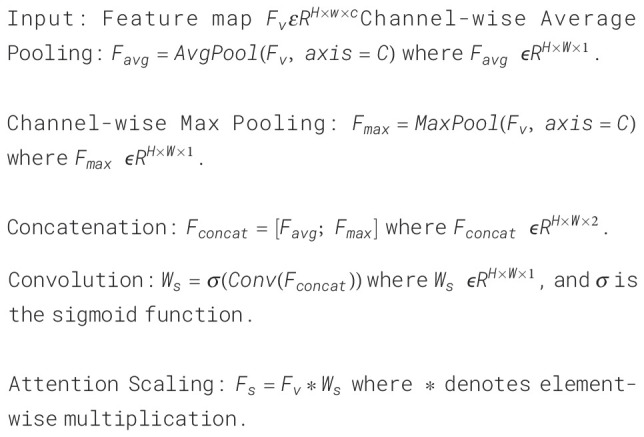

### 3.2 Audio feature extraction

**Figure 6:**
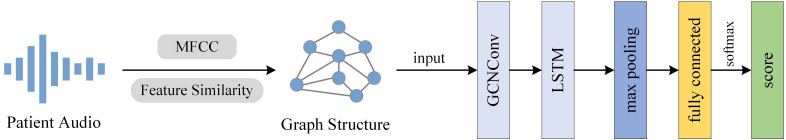
Audio feature extraction.

The researchers found that people with depression tended to speak in a monotonous and lower tone than healthy controls (21). Therefore, in addition to the analysis of visual features, it is particularly important to mine the key features hidden in speech signals for the diagnosis of depression. See **Figure 6** for the audio feature processing method used in this paper.

We use Mel-frequency Cepstral coefficients (MFCC) of audio data as effective features for depression recognition (22). MFCC takes into account the auditory properties of the human ears and can well capture the features in speech. The calculation process of MFCC is as follows: Firstly, the input audio signal is pre-weighted to enhance the high-frequency components. Then, the pre-weighted signal is divided into multiple short-time frames, and a window is applied to each frame to reduce the spectral leakage. Next, the fast fourier transform is performed on each windowed frame to convert the time domain signal to the frequency domain. Then the power spectrum of each frame is calculated. Finally, the power spectrum is passed through a bank of Mel filters, and its output is log transformed and discrete cosine transformed. At this point, we have the MFCC feature vector for each frame of the input audio. After that, we consider the MFCC feature vector of each frame as a node, and calculate the feature similarity of each node based on the Euclidean distance between its feature vectors, so as to construct the edges between nodes. In this process, we set a threshold of 0.5 to limit the addition of edges, that is, only adding edges between nodes with high enough feature similarity and small enough distance. Finally, we assign a corresponding weight to each edge based on the inverse of the distance to better capture the local and global features in the audio signal. The specific algorithmic architecture of MFCC Calculation is shown in **Algorithm 3**.

Algorithm 3 **Pseudocode of MFCC calculation.**

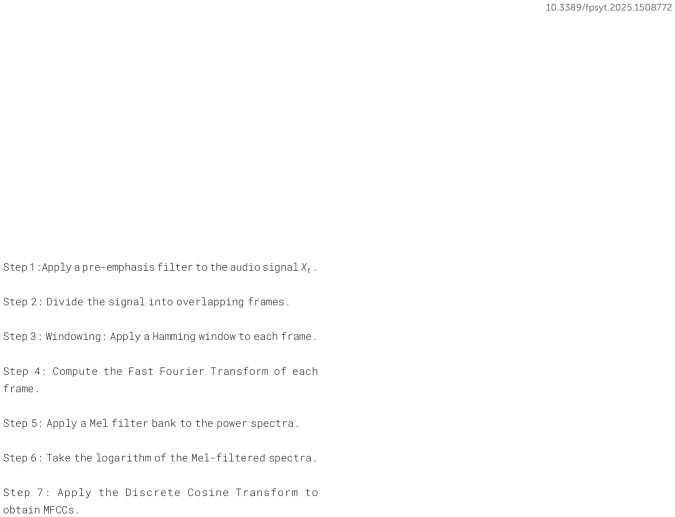

To explore the complex patterns and temporal features of audio data, we input the constructed graph structure data into GCN and LSTM for processing. GCN aggregates the features of nodes and their neighbors through convolution operations. LSTM could capture long and short-term dependencies in the sequence. **Algorithm 4** further details the process of combining GCN and LSTM. By combining GCN and LSTM, we can capture the high-level graph features of each node and the dynamics and dependencies in the time series. Finally, by subsampling and classifying the complex features, we can obtain a diagnosis of whether the speaker in the audio has depression or not.

Algorithm 4 **Pseudocode of GCN-LSTM model.**

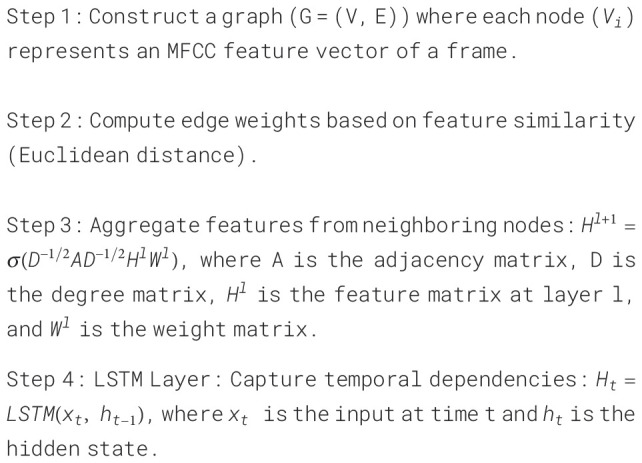

### 3.3 Video-audio fusion

**Figure 7:**
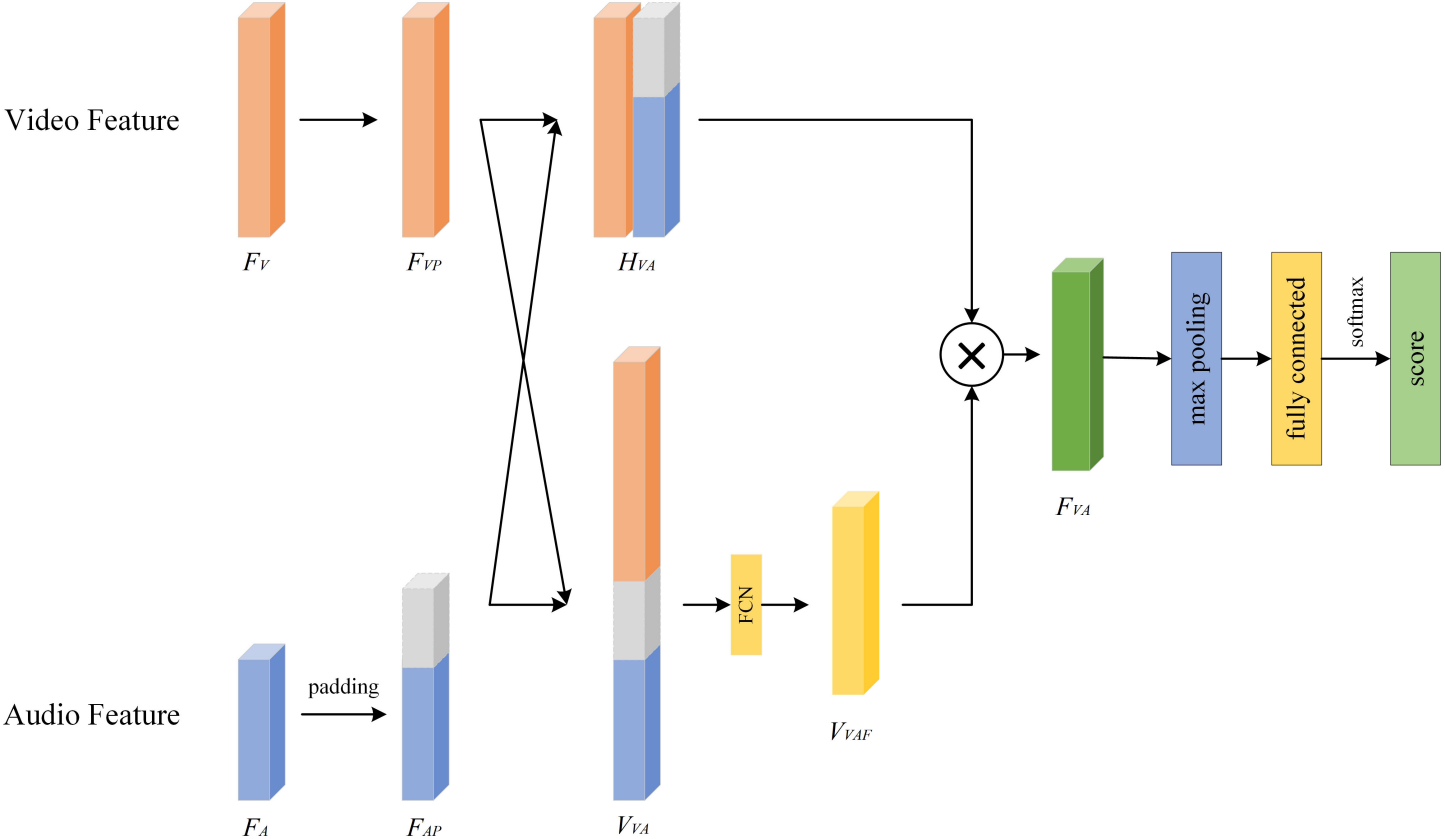
Video-audio fusion network.

Assuming that the final extracted visual feature is $F_{V}$ and the obtained audio feature is $F_{A}$ , we now discuss how to fuse these two features. In view of the fact that not all modality features play a positive role in the severity assessment of depression, we propose a Video-Audio Fusion Network (VAFN) to fuse the feature information of the two modalities. The structure of VAFN is shown in **Figure 7**. The input of VAFN is the multi-modal feature $F_{MM}=\left\{F_{V},F_{A}\right\}$ , and the output feature is the fused $F_{VA}$ . The nature of video and audio data are different, which leads to different feature vector dimensions. Therefore, in the actual processing, we first perform zero-padding on the side with smaller size in $F_{V}$ and $F_{A}$ to ensure that the resulting dimensions of $F_{VP}$ and $F_{AP}$ are consistent. Then, $F_{VP}$ and $F_{AP}$ are superimposed in the horizontal and vertical directions respectively to obtain $H_{VA}$ and $V_{VA}$ . A fully connected layer is used to reduce the dimension of $V_{VA}$ , and the attention weight vector $V_{VAF}$ is obtained. Finally, $H_{VA}$ and $V_{VAF}$ are multiplied to obtain the final multi-modal fusion feature $F_{VA}$ . The obtained fusion features are max-pooling and classified to obtain the depression prediction results. The entire fusion process described above is summarized in **Algorithm 5**.

Algorithm 5 **Pseudocode of VAFN.**

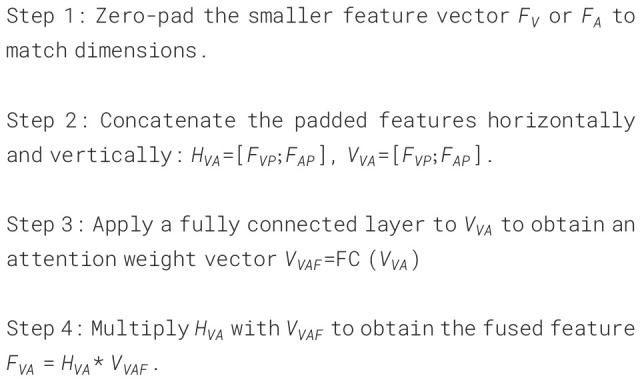

## 4 Experiments

### 4.1 Experimental settings

The GPU used in this paper is NVIDIA RTX3090. The development and testing are carried out in the Python3.9 environment, and the integrated development tool is Pycharm. We use PyTorch v1.12.0 as the deep learning framework and use CUDA 11.6 in the model training process. The operating system is Windows10. In order to alleviate the over-fitting problem, we use the AdamW optimizer for training, and add the Dropout layer to the network backbone. The dropout rate is set between [0.4, 0.6]. As for optimization, the learning rate is set to be 10e−4 for modality feature extraction and 5×10e−5 for modality fusion with linear schedule strategy. During training, the number of iterations is consistently set to 5000.

In this study, we used the Extended Distress Analysis Interview Corpus (E-DAIC) dataset (23, 24) to validate our proposed method. The E-DAIC dataset is an extended version of the WOZ-DAIC dataset (23) and consists of semi-structured clinical interviews designed to identify psychological distress conditions such as anxiety, depression, and PTSD. In E-DAIC dataset, OpenSMILE (25) was used to extract the acoustic features of subjects, including Mel-frequency cepstral coefficients (MFCC) (26), and OpenFace (27) was used to extract the corresponding visual features. Facial features, eye fixations, head poses, and motor units are included. To protect the privacy of participants, the dataset provides these extracted features directly instead of raw video recordings. The E-DAIC dataset consists of clinical interview transcripts from 219 participants, along with corresponding assessments of depression and PTSD severity. To ensure a representative distribution of the data, the training set contains 163 samples, the validation set contains 56 samples, and the test set contains 10 samples. Each participant in the E-DAIC dataset was annotated according to their Patient Health Questionny-8 (PHQ-8) score (28), with scores higher than 10 classified as 1 (indicating the presence of depression) and scores lower than 10 labeled as 0 (indicating the absence of depression).

### 4.2 Evaluation metrics

Evaluation measures to evaluate the depression diagnostic model included F1 score (Equation 7) (29), root-mean-square error (RMSE, Equation 8) and mean absolute error (MAE, Equation 9) (30). The F1 score is the harmonic average of precision and recall (Equation 6) and is used to comprehensively measure the performance of the depression diagnostic model. RMSE can reveal how the model performs in extreme cases, such as severely overestimating or underestimating a patient"s depression, which can have a significant impact on clinical decision making. MAE gives the average difference between the model prediction and the actual value.


(6) $$Precision=\frac{\text{T}P}{\text{T}P+FP},\;Recall=\frac{\text{T}P}{TP+FN}$$

(7) $$F1\;=\;2\times \frac{\text{Pr}ecision\times Recall}{\text{P}recision+Recall}$$

(8) $$RMSE=\sqrt{\frac{1}{N}\sum_{i=1}^{N}{\left(y_{i}-{\hat{y}}_{i}\right)}^{2}}$$

(9) $$MAE=\frac{1}{N}\sum_{i=1}^{N}\left|y_{i}-{\hat{y}}_{i}\right|$$

### 4.3 Results

#### 4.3.1 Comparison with other methods

In this study, we preliminarily use video data and audio data separately for depression recognition based on single-modal features, and the results are shown in **Table 1**. Specifically, for video features, we compare the proposed TSNet-DD with references (6, 10, 12). It shows that TSNet-DD consistently outperforms the other three models, and TSNet64-DD outperforms TSNet32-DD. The TSNet64-DD model achieved the highest F1 score of 0.853, demonstrating its ability to capture both spatial and temporal features effectively. This improvement over previous models (6, 10, 12) suggests that our temporal-spatial attention mechanism significantly enhances feature extraction. Although the RMSE value of TSNet64-DD is slightly higher than that of TSNet32-DD at 5.11, the small difference in RMSE here is negligible compared to the advantages of its F1 value and MAE value. For audio data, the GCN-LSTM model achieved an F1 score of 0.827, outperforming previous models (13) and (14). This indicates that combining GCN and LSTM can effectively capture the complex patterns in audio features related to depression. Although the MAE value of GCN-LSTM is not the lowest, considering the characteristic that MAE is insensitive to outliers and its excellent performance on F1, we believe that GCN-LSTM has a unique advantage in processing audio features of depression.

**Table 1:** Results of depression recognition under single-modal features.

feature	model	F1	RMSE	MAE
video	RNN-C3D (6)	0.723	8.07	5.78
	STA-DRN (10)	0.702	8.94	6.77
	RFR (12)	0.710	8.49	6.57
	TSNet32-DD	0.800	5.11	5.03
	TSNet64-DD	0.853	5.23	4.45
audio	GCNN (13)	0.690	9.28	6.65
	GLNN (14)	0.788	8.43	5.04
	GCN-LSTM	0.827	6.67	6.28

Subsequently, we fuse facial video features and audio features to evaluate the performance of multimodal data in depression diagnosis. The results are shown in **Table 2**. We find that the model based on multi-modal data consistently outperforms the performance using only single-modal data in terms of F1 value. The F1 value of our proposed method finally reaches 0.922, which is not only better than the performance of all single-modal data, but also the highest in all experiments based on multi-modal data. This may be due to the diversity of the input data, and it indicates that there are clear differences in facial visual features and voice features between patients with depression and healthy participants. It turns out that the multimodality-based assistive method has its unique significance in depression diagnosis when the privacy of the participants is protected as much as possible.

**Table 2:** Results of depression recognition under multi-modal features.

model	F1	RMSE	MAE
MFM-Att (17)	0.895	7.29	4.03
GCN	0.918	6.24	3.88
VAFN(TSNet32+GL)	0.903	5.77	4.00
VAFN(TSNet64+GL)	0.922	6.06	3.51

**Figure 8:**
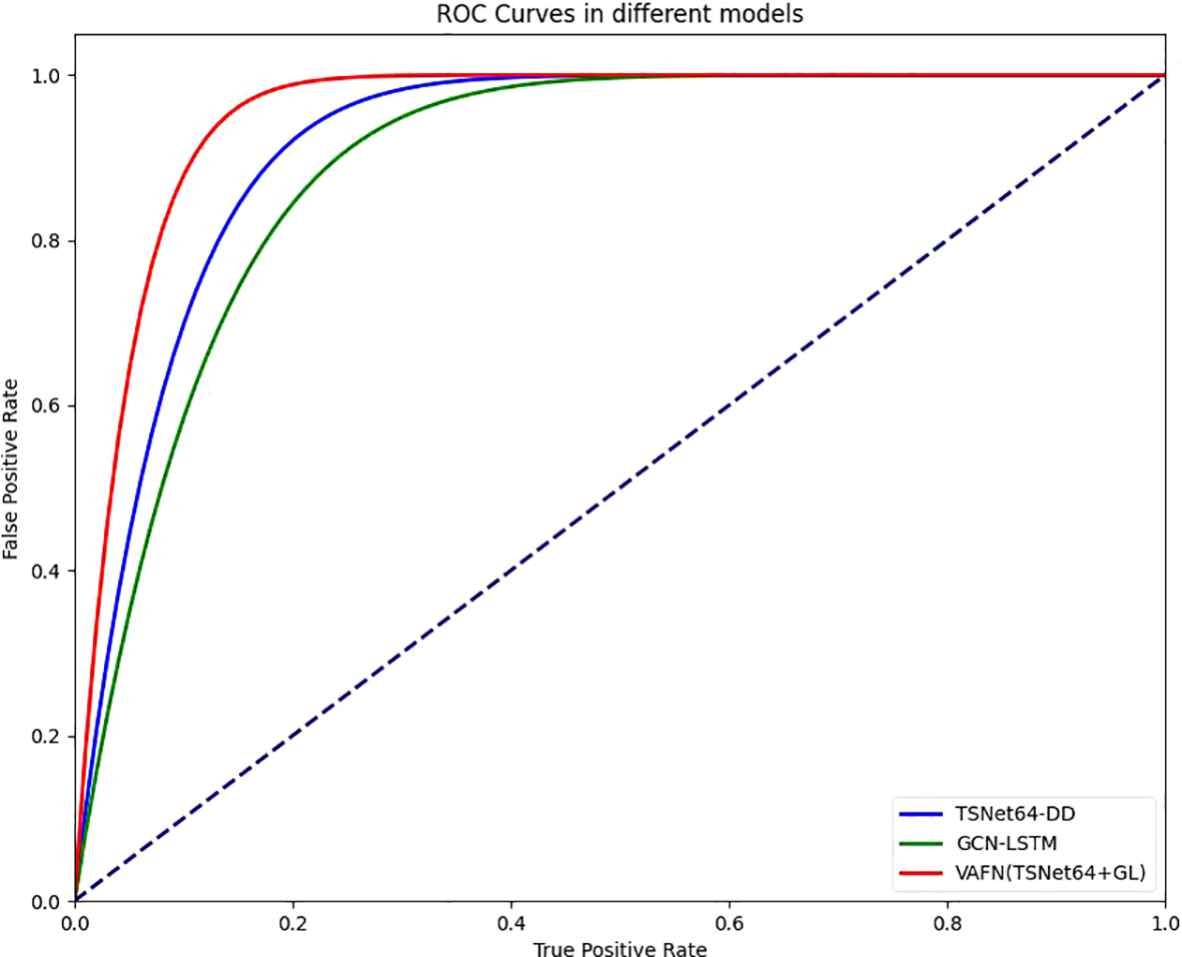
Comparison of different ROCs.

To provide a comprehensive evaluation of our model’s performance, we also included the Receiver Operating Characteristic (ROC) curve and the Area Under the Curve (AUC) values. The ROC curve is a graphical representation that illustrates the diagnostic ability of a binary classifier system as its discrimination threshold is varied. The AUC provides an aggregate measure of performance across all possible classification thresholds. Our model’s ROC curves for both single-modal and multi-modal data are shown in **Figure 8**. The AUC values for TSNet64-DD, GCN-LSTM, and VAFN(TSNet64+GL) are summarized in **Table 3**. The results indicate that our multi-modal fusion model achieves the highest AUC value, further confirming its superior performance in distinguishing between depressed and non-depressed individuals.

**Table 3:** Results of different model’s AUC values.

model	AUC	FLOPs
TSNet64-DD	0.912	453,787,648
GCN-LSTM	0.880	4,915,200
VAFN(TSNet64+GL)	0.950	463,424,512

In addition, our proposed models strike a balance between computational complexity and performance. The computational complexity of TSNet-DD is primarily determined by the number of convolutional layers and the attention modules. The attention modules add a small overhead due to the additional operations for attention weight calculation. Despite the added complexity of the attention modules, TSNet-DD is designed to be non-redundant, ensuring efficient processing without unnecessary computational overhead. This balance between complexity and efficiency allows TSNet-DD to achieve high performance while maintaining reasonable computational requirements. GCN-LSTM combines the strengths of GCN and LSTM to process audio features. GCN is used to aggregate features from neighboring nodes, while LSTM captures temporal dependencies. GCN-LSTM is designed to handle the variability and noise in audio data effectively. While the combination of GCN and LSTM increases the computational complexity, the model’s ability to capture complex patterns and temporal features justifies the additional computational cost. VAFN fuses the feature information from both video and audio modalities. It uses zero-padding to handle different feature vector dimensions and attention mechanisms to assign adaptive weights to the features. The overall complexity of VAFN depends on the individual complexities of TSNet-DD and GCN-LSTM, along with the additional operations for feature fusion. The fusion process involves concatenation, fully connected layers, and attention weight calculation, which add to the computational load. VAFN is designed to leverage the complementary nature of visual and audio features, resulting in improved diagnostic performance. The fusion process, while adding some computational overhead, is optimized to ensure that the model remains efficient and scalable.

#### 4.3.2 Ablation study

To better understand the contributions of various components in our proposed model, we conducted an ablation study. This study evaluates the impact of the Temporal-Spatial Attention Module (TSAM), the combination of Graph Convolutional Network (GCN) and Long Short-Term Memory (LSTM) for audio features, and the Video-Audio Fusion Network (VAFN). We performed experiments by systematically removing or modifying these components and observing the resulting changes in performance metrics.

We first assess the effect of the TSAM by comparing the full TSNet64-DD model with a variant that does not include the TSAM. The results are shown in **Table 4**. It demonstrates that the TSAM significantly improves the performance of the model, highlighting its importance in capturing temporal and spatial features. Next, we evaluate the impact of using GCN and LSTM for audio feature extraction. We compare the full GCN-LSTM model with variants that use only GCN or only LSTM. The results are shown in **Table 5**. The results indicate that the combination of GCN and LSTM outperforms the individual models, demonstrating the effectiveness of integrating both graph-based and temporal features for audio data. Finally, we assess the impact of the VAFN by comparing the full VAFN(TSNet64+GL) model with variants that use only video features (TSNet64-DD) or only audio features (GCN-LSTM). The results are shown in **Table 6**. The results clearly show that the fusion of video and audio features significantly enhances the performance, confirming the complementary nature of these modalities. The ablation study confirms that each component of our proposed model contributes to its overall performance. The TSAM enhances the extraction of temporal and spatial features from video data, the combination of GCN and LSTM effectively captures complex audio patterns, and the VAFN successfully integrates multimodal features to improve diagnostic accuracy. These findings validate the design choices and highlight the importance of multimodal data fusion in the automatic diagnosis of depression.

**Table 4:** Impact of Temporal-Spatial Attention Module (TSAM) on video feature extraction.

Model	F1	RMSE	MAE
TSNet64-DD	0.853	5.23	4.45
TSNet64-DD w/o TSAM	0.789	6.12	5.37

**Table 5:** Impact of GCN and LSTM on audio feature extraction.

Model	F1	RMSE	MAE
GCN-LSTM	0.827	6.67	6.28
GCN only	0.742	7.89	7.12
LSTM only	0.756	7.65	6.87

**Table 6:** Impact of Video-Audio Fusion Network (VAFN) on multimodal feature fusion.

Model	F1	RMSE	MAE
VAFN(TSNet64+GL)	0.922	6.06	3.51
TSNet64-DD	0.853	5.23	4.45
GCN-LSTM	0.827	6.67	6.28

#### 4.3.3 Effects of different subject groupings

**Figure 9:**
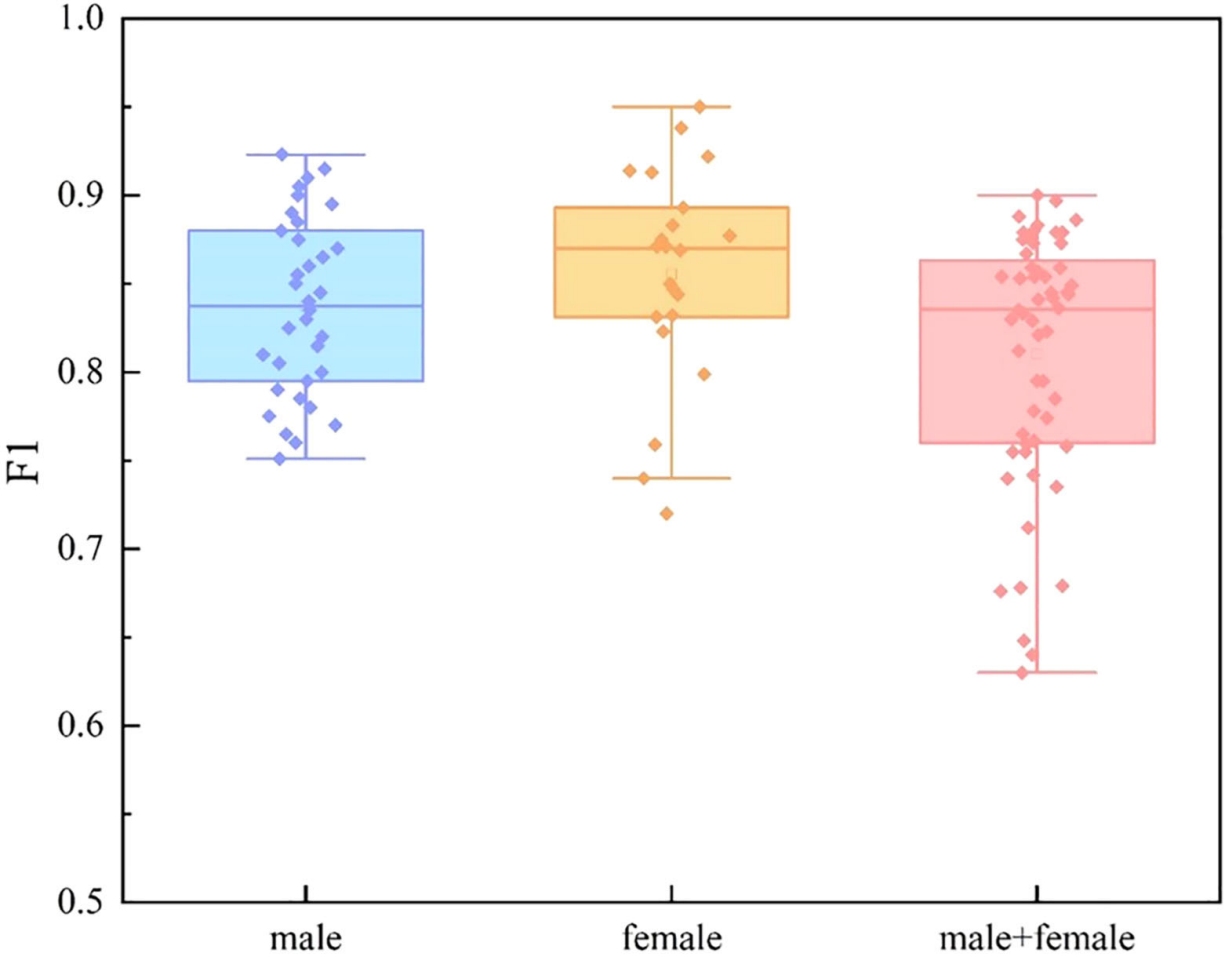
Comparison of different subject groups on video modal features.

**Figure 10:**
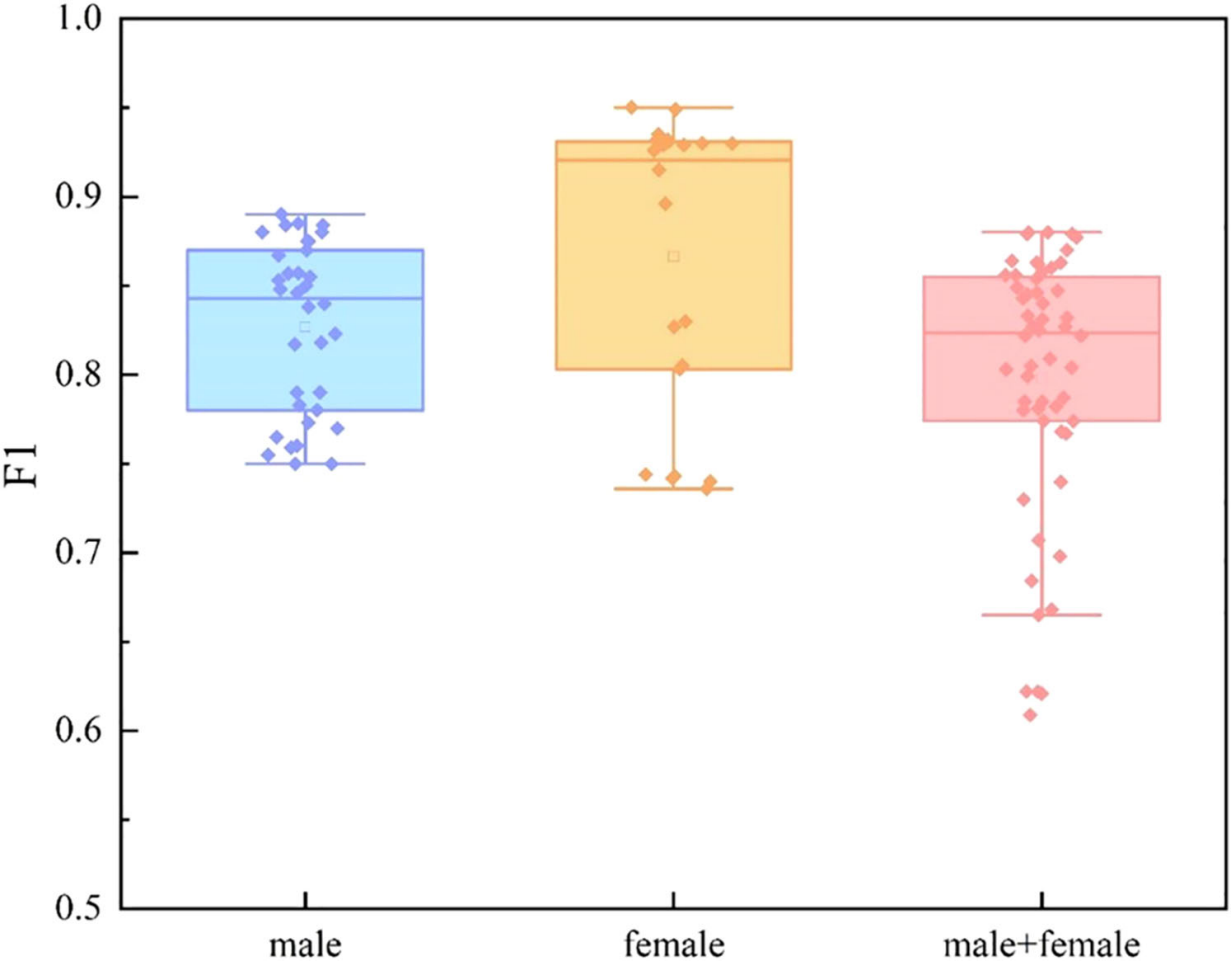
Comparison of different subject groups on audio modal features.

**Figure 11:**
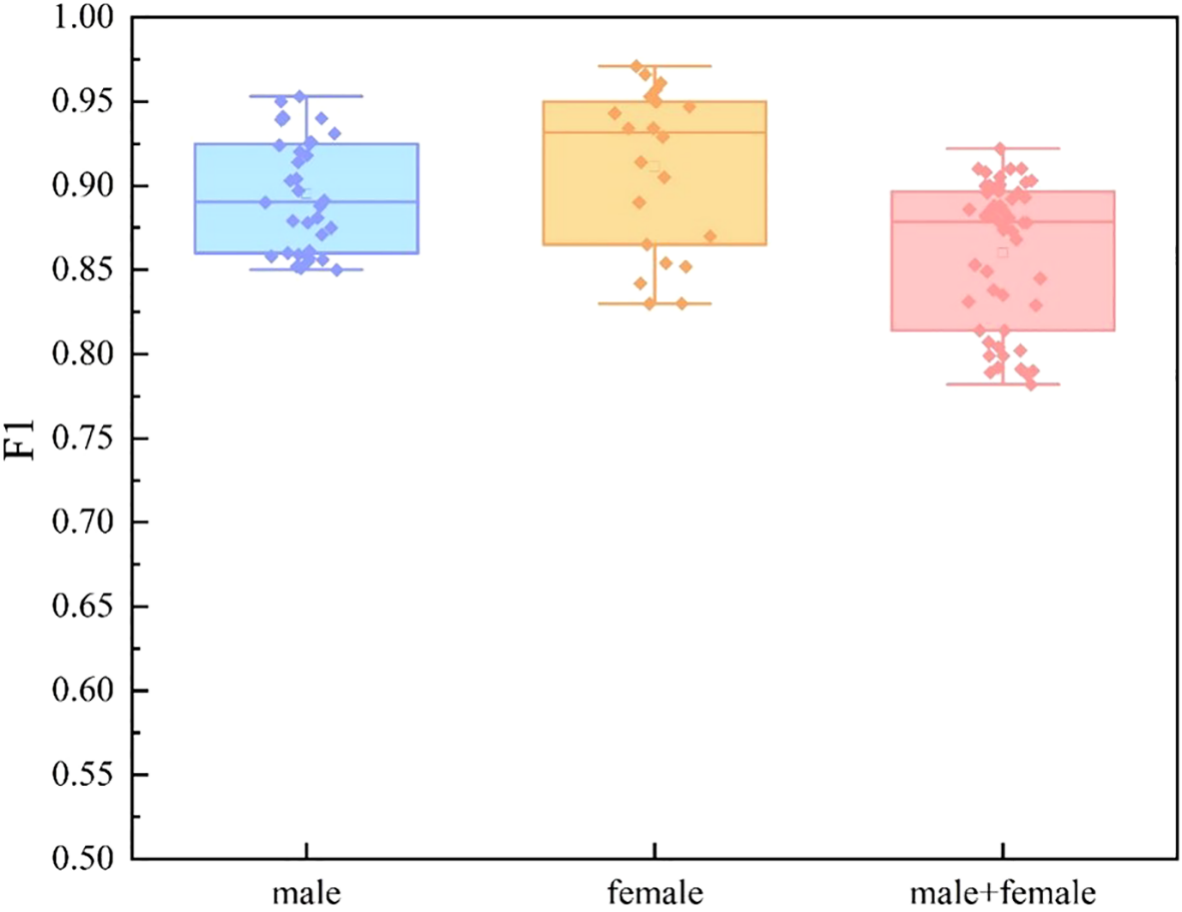
Comparison of different subject groups on multimodal features.

We divide the dataset into three categories by sex: male group, female group, and mixed gender group. For each data set, we conducted experiments based on single mode and multi-mode respectively. In the video mode experiment, we adopt TSNet64, which has better performance. The experimental results are shown in **Figure 9**, **Figure 10** and **Figure 11**. We find that for each modal, the F1 values of the mixed gender group are consistently lower than those assessed on either the male or female group alone. In addition, the female group almost always outperformed the male group, which may be due to a category imbalance between the samples. It also suggests that men and women differ in the information conveyed in facial behavior and speech during the diagnosis of depression. In addition, for each subject group, the experimental results based on the multimodal feature set are generally better than those based on the single-modal feature set, which is mainly due to the diversity of training data which brings more abundant features.

### 4.4 Interpretability analysis

**Figure 12:**
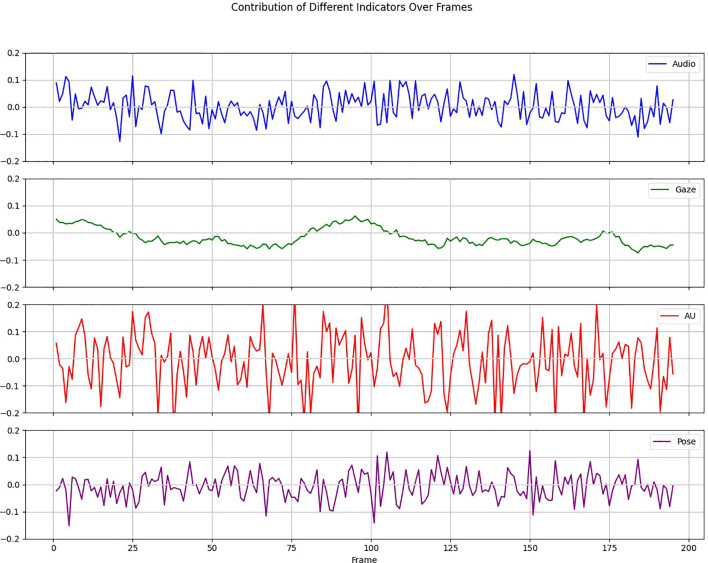
Contribution of different indicators over frames.

**Figure 13:**
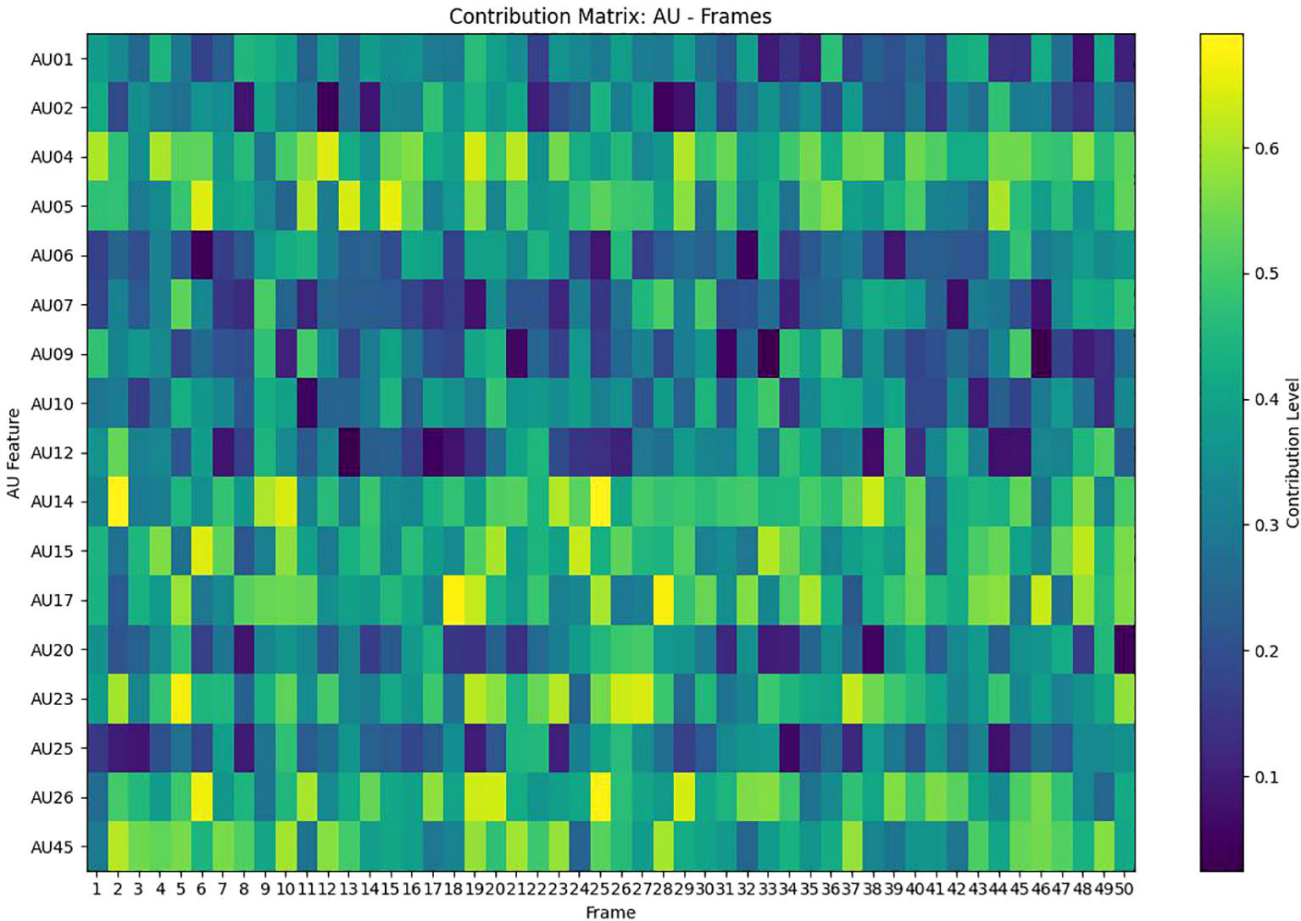
Contribution matrix of AU and frames.

Deep learning techniques are usually ‘black-box’, but in clinical practice we need more transparent models to increase their credibility and interpretability. Therefore, we perform an interpretability analysis of our model. We show the attribution scores for audio, gaze, action unit (AU), and pose, where higher values indicate strong attribution to positive predictions. The E-DAIC dataset has more than 19,000 frames of facial images for each sample, and we aggregate every 100 frames into a whole to explain depression detection. As shown in **Figure 12**, AU contributes to the model diagnostic results to the highest degree, followed by pose and audio, and the smallest contribution is gaze. We further visualize the degree of influence of each AU feature on the diagnostic results of different frames through the contribution matrix. As can be seen in **Figure 13**, AU04, AU05, AU14, AU15, AU17, AU23, AU26, and AU45 have a higher degree of influence, indicating a stronger correlation with depression. These units thus play a more significant role in the diagnostic process for depression.

### 4.5 Discussions

This study introduces a novel multi-modal deep convolutional network that leverages multi-source data fusion to provide a more effective solution for the automatic diagnosis of depression. We utilize a feature fusion module to effectively integrate temporal and spatial features, thereby extracting a more comprehensive representation that is conducive to analyzing the psychological state of patients. Our model’s complexity is balanced by its non-redundant design, ensuring efficient processing without unnecessary computational overhead. From our comprehensive test results, several noteworthy insights can be gathered. The proposed TSNet-DD model for video data demonstrates significant advantages in capturing both spatial and temporal features. For audio data, the GCN-LSTM model effectively captures complex patterns related to depression. The fusion of video and audio features further improves diagnostic performance, demonstrating the complementary nature of visual and audio features.

However, it is important to acknowledge some limitations inherent in our study. First, due to the lack of publicly available high-quality datasets in this field, our research focuses specifically on E-DAIC datasets. Future research should aim to extend this approach to include different datasets for various psychiatric disorder diagnoses. Additionally, it is important to categorize different levels of depression, which we plan to address in future studies. Furthermore, inspired by ACFun (31) and LMAC-ZS (32), future research could explore the integration of additional data types, such as textual information, into the model to enhance classification performance. This integration could provide the model with a more comprehensive contextual understanding, thereby improving its ability to recognize emotional states. Considering the inherent limitations of deep learning—black-box—we can draw from the methodologies proposed in LMAC-ZS to enhance our model’s interpretability. This kind of interpretability mechanism not only contributes to transparency in clinical applications but also provides significant directions for future research.

Overall, our results underscore the importance of multi-modal data in improving the accuracy and reliability of depression diagnosis. Future work should focus on expanding the dataset to include more diverse populations and exploring additional modalities such as text or physiological signals to further enhance diagnostic capabilities.

## 5 Conclusion

On the premise of protecting the privacy of patients, this paper discusses the method of realizing the high precision diagnosis of depression. We design TSNet-DD architecture for video data to comprehensively consider the temporal and spatial features of video frames through the spatial-temporal attention mechanism module. For audio data, we use a combination of GCN and LSTM to capture high-level graph features and dynamic changes in timing. Finally, the multi-modal feature fusion is realized through the video-audio fusion network. The experimental results show that our method has certain potential in the automatic diagnosis of depression. In the future, researchers can further explore the sensitive features of automatic recognition of depression through larger data sets and more diverse modalities, so as to improve the recognition accuracy and provide more powerful diagnostic and treatment support for clinicians. In addition, it is crucial to classify different levels of depression, which we plan to address in future studies.

## Data Availability

Publicly available datasets were analyzed in this study. This data can be found here: https://dcapswoz.ict.usc.edu/extended-daic-database-download/ (USC).
